# Prevalence estimation of intellectual disability using national administrative and household survey data: The importance of survey question specificity

**DOI:** 10.23889/ijpds.v6i1.1342

**Published:** 2021-01-28

**Authors:** O McBride, P Heslop, G Glover, T Taggart, L Hanna-Trainor, M Shevlin, J Murphy

**Affiliations:** School of Psychology, Ulster University, Coleraine, Northern Ireland; School for Policy Studies, University of Bristol, Bristol, United Kingdom; Learning Disability Observatory, Public Health England, London, United Kingdom; School of Nursing, Ulster University, Derry, Northern Ireland

**Keywords:** intellectual disability, census data, administrative data, household probability survey, northern ireland

## Abstract

**Background:**

Variability in prevalence estimation of intellectual disability has been attributed to heterogeneity in study settings, methodologies, and intellectual disability case definitions. Among studies based on national household survey data specifically, variability in prevalence estimation has partly been attributed to the level of specificity of the survey questions employed to determine the presence of intellectual disability.

**Specific aims & method:**

Using standardised difference scoring, and ‘intellectual disability’ survey data from the 2007 Northern Ireland Survey on Activity Limitation and Disability (NISALD) (N=23,689) and the 2011 Northern Ireland Census (N=1,770,217) the following study had two aims. First, we aimed to demonstrate the effects of survey question specificity on intellectual disability prevalence estimation. Second, we aimed to produce reliable estimates of the geographic variation of intellectual disability within private households in Northern Ireland while also assessing the socio-demographic, health-related and disability characteristics of this population.

**Findings:**

Prevalence estimates generated using the more crudely classified intellectual disability Census data indicated a prevalence of 2% for the overall population, 3.8% for children aged between 0 and 15 years, and 1.5% for citizens aged 16 years or older. Intellectual disability prevalence estimates generated using the more explicitly defined 2007 NISALD data indicated a population prevalence of 0.5% for the overall population, 1.3% for children aged between 0 and 15 years, and 0.3% for citizens aged 16 years or older. The NISALD estimates were consistent with most recent international meta-analysis prevalence estimates. According to the NISALD data, the majority of those with an intellectual disability were male, lived outside Belfast, and experienced severe intellectual disability, with multiple comorbid health conditions.

**Discussion:**

The current findings highlight the importance of survey question specificity in the estimation of intellectual disability prevalence and provide reliable prevalence estimates of intellectual disability in Northern Ireland. The findings also demonstrate the utility of administrative data for detecting and understanding intellectual disability, and inform recommendations on how to maximise use of future intellectual disability Census data

## Introduction

According to most recent meta-analysis findings, the prevalence of intellectual disability ranges from 0.05 – 1.55% globally [1]. Reviewing 20 studies (from Australia, Canada, China, Denmark, Finland, India, Norway, Taiwan & Sweden), McKenzie and colleagues showed that intellectual disability prevalence was highly variable. Estimates based on studies using child/adolescent data ranged from 0.22 % to 1.55 % [2, 3] while estimates based on studies using adult data ranged from 0.05 % to 0.8 % [4, 5]. Estimates based on data that included both children/adolescents and adults ranged from 0.10 % to 1.30 % [6, 7]. Regarding prevalence over time, seven studies provided estimates across multiple years [2, 8-13]. Of these, one study revealed an increase over time [11], three reported decreasing prevalence [8, 12-13], while three identified no time trend [8, 10, 2].

The authors partly attributed this variability in prevalence to heterogeneity in study settings, methodologies, and case definitions. Specifically, studies tended to vary in data source i.e. administrative data (e.g. health, education, social services data or data from national registries) or national household survey data. Moreover, administrative data-based studies were distinct from studies based on national household survey data in relation to intellectual disability classification. Classification of intellectual disability in administrative data-based studies tended to be determined by clinical diagnosis, a recognized classification system (ICD; DSM; AAMR), psychological assessments, use of intellectual disability services, legal definition, and/or receipt of special education. However, intellectual disability classification in studies based on national household survey data tended to be based on survey questions (all slightly different) designed to ascertain the presence of intellectual disability.

Notably, variation in the format and content of these survey questions, particularly in studies based in the same country and administered during the same time, returned varying intellectual disability prevalence estimates. For instance, where intellectual disability prevalence estimates were derived from adult participant responses to two separate Canadian national household surveys that used different questions to ascertain the presence of intellectual disability, prevalence varied from 0.2% in one survey (survey question = ‘*Do you have autism or any other developmental disorder such as Down’s syndrome, Asperger’s syndrome, or Rett syndrome*’) to 0.5% in the other (survey question = ‘*Has a doctor, psychologist or other health professional ever said that you had a developmental disability or disorder (examples provided)*’) [14]. Moreover, where intellectual disability prevalence estimates were generated using child/adolescent participant data from two separate longitudinal US national household surveys (1997 – 2008; 2001 – 2011) that also used different questions to ascertain the presence of intellectual disability, prevalence varied from less than 0.4% in one study (survey question = ‘*Has a doctor or health professional ever told you that [survey child] has any of the following conditions? (included—autism, mental retardation)*’ [2] to greater than 0.6% in the other (survey question = ‘*What caused disability/limitation? (options included mental retardation)*’ [10].

While poorer classification of intellectual disability has notable effects on prevalence estimation, it has also been shown to dramatically influence assessments of related morbidity. For instance, Lin and colleagues [5] employed three algorithms (broad, intermediate & narrow) to vary the sensitivity/specificity of intellectual disability classification. They found that using the narrow algorithm classified substantially more individuals with psychiatric co-morbidities than using the intermediate algorithm. These findings provided useful evidence of how intellectual disability mis/classification might disrupt or compromise our assessment of other factors that are important in attaining a clearer understanding of the epidemiology of intellectual disability. Given that national administrative and household panel survey data are widely used to inform prevalence estimates of intellectual disability, our study used two separate sources of national survey data from Northern Ireland to investigate the effects of survey question ambiguity/specificity on intellectual disability prevalence estimation. We used 2011 Census data from Northern Ireland, that contained citizen responses to specific health condition categories. One of these, crudely specified intellectual disability (Do you have any of the following conditions which have lasted, or are expected to last, at least 12 months? ‘A learning difficulty, an intellectual difficulty, or a social or behavioural difficulty’). We also used data from the 2007 Northern Ireland Survey on Activity Limitation and Disability (NISALD) [15]. This asked participants to separately report the presence of ‘a learning difficulty’, ‘an intellectual difficulty’ or ‘a social or behavioural difficulty’. See Table 1 for question content comparisons between surveys.

Our study had four objectives:

First, we sought to compare the sex, age, geographic, and health-related distributions of the overall samples from both surveys. We did this (i) to demonstrate the comparability of the data from both surveys and (ii) to assess whether comparisons based on survey content that varied between surveys (i.e. highly variable health condition survey content) were less reliable than comparisons based on content that was identical between surveys (e.g. sex, age, geographical location).

Second, we sought to compare the prevalence estimate of intellectual disability generated from the 2011 Census data with the prevalence estimate of intellectual disability generated from the NISALD data to assess how survey item specificity affects intellectual disability prevalence estimation.

Third, we sought to compare the sex, age, geographical, economic-activity and health-related distributions of the intellectual disability subsamples from both surveys to assess whether the adoption of a cruder classification of intellectual disability would influence the distribution and comparison of these variables.

Finally, we sought to report the socio-demographic and health-related characteristics of those individuals who endorsed the intellectual difficulty item from the 2007 NISALD survey. In doing this, we wanted to profile the Northern Irish intellectual disability population using the more explicit and specific descriptor of intellectual disability, and, in so doing, provide a benchmark against which the more explicit 2021 Census data relating to intellectual disability can be measured in the future.

## Method

### Data sources

#### 1. 2007 Northern Ireland Survey of people with Activity Limitations and Disability (NISALD)

The NISALD [15] was conducted by the Northern Ireland Statistics and Research Agency (NISRA) during 2006-2007. The survey aimed to provide an up-to-date, accurate picture of the prevalence and circumstances of adults and children living with a disability in Northern Ireland. Although the NISALD comprised of two strands i.e. (1) private households and (2) communal establishments (excluding places of detention and military establishments), only data and findings from the private household strand have been released publicly and, as such, the current study relates to disability and activity limitations within *private households* in Northern Ireland only.

There was no comprehensive register of people with disabilities in Northern Ireland (i.e. no sampling frame) from which to sample potential respondents for the NISALD survey. To overcome this obstacle, NISRA selected a random sample of 12,000 households from the Northern Ireland Valuation and Lands Agency Database, which contains a record of all domestic households in Northern Ireland, to serve as the study sampling frame. Selected households were posted a letter in advance which contained detailed information about the NISALD, its purpose, and that their household would be contacted in due course with respect to potential participation. Of the initial sample of 12,000 households, 10,984 (84% of random sample) were eligible addresses. Subsequently, NISRA contacted each eligible household to conduct a screening exercise with one member of the household (largely via telephone, but also via face-to-face interview if requested or if the household did not have a telephone) during which information was requested on each member living in the household. Information sought included basic demographic information on each member of the household, as well as the presence and level of difficulty associated with 15 disabilities or activity limitations (in accordance World Health Organisation (WHO) International Classification of Functioning, Disability and Health (ICF) 16) that had lasted or were expected to last **six months** (see Table 1).

In total, 23,689 screening interviews were conducted with 18,517 adults and 5,172 children in eligible households. Individuals within the household who reported: (1) more than one mild limitation that affected activities sometimes; (2) at least one mild limitation that affected activities often; or (3) one or more moderate/severe limitation (n=4,185; 3,865 adults and 320 children), were considered as having some medical, social or environmental factor that affected their ability and therefore were deemed eligible to complete a questionnaire designed to further assess the nature of their activity limitations and disability.

Multiple members of the same household could complete this questionnaire if they met the eligibility criteria, which was conducted via face-to-face interviewing in the respondent’s own home. Adult and child versions of the main questionnaire were largely similar with only minor amendments to wording for children. All interviews for respondents aged 15 years or younger were conducted in proxy form with the parent or guardian.

The main questionnaire collected information on: how the disability or activity limitation affected daily life; use of aids, specialised equipment or medication; management of disabilities; supports and care needed/received; general health; use of health and social care services; education; employment and training; social participation, leisure and attitudes of others; transport and travel; housing; crime and fear of crime; additional costs relating to living with a disability (e.g. goods, services, equipment or medication), income and benefits; and general background information. Approximately 85% of those who were eligible and invited to participate in the main interview did so (n=3,543; 3,262 adults and 281 children). Data from NISALD is available for researchers to access via the UK Data Service, study number 7236 (https://beta.ukdataservice.ac.uk/datacatalogue/studies/study?id=7236).

#### 2. 2011 Census

The Census aimed to collect information on all residents of Northern Ireland (N=1,744,966) living in both private households (N=1,723,942) and communal establishments (N=21,024). Census data was obtained from 1,723,180 residents (98.8% of entire population), of which 1,702,217 lived in private households and 20,963 lived in communal establishments. Because communal establishment data was unavailable for the NISALD survey, it has been excluded from the analysis of Census data in this study. The Census form indicated that the householder (the person who owned or rented the accommodation and was responsible for paying the household bills) was responsible for ensuring that the questionnaire was completed and returned, although it did not provide instructions as to who should complete the separate sections of the Census form relating to each individual household member. It was generally assumed, however, that parents would have completed the individual forms on behalf of younger children while older children would have self-reported. In the 2011 Census, individuals were asked whether they had any of a list of health conditions (see Table 1) which had lasted or were expected to last, at least **twelve months**. There was also an option to report no health conditions.

Key differences in the methodologies of the 2007 NISALD and the 2011 Census in relation to measuring the prevalence of disability and other health conditions in Northern Ireland are summarised in Table 1. For the purposes of this investigation, the most notable difference between the two sources related to the separation of intellectual difficulty from learning difficulty and social or behavioural difficulty in the NISALD, compared to the aggregation of these three conditions in the 2011 Census. Thus, the NISALD presented a *unique* opportunity to explicitly identify those individuals who had an intellectual difficulty or developmental delay.

**Table 1: Overview of the content, phrasing, and mode of administration of questions used to ascertain the presence of disability and other health conditions in the 2007 Northern Ireland Survey on Activity Limitations and Disability and the 2011 Northern Ireland Census:** 

	2007 Northern Ireland Survey on Activity Limitations and Disability	2011 Census

Method of administration	(telephone and face-to-face interview)	(paper based self-report)
Introductory statement/question	The survey is only interested in difficulties or activity limitations that have lasted or are expected to last 6 months or more. Do you/any of the people in your household have any difficulty…
	Do you have any of the following conditions which have lasted, or are expected to last, at least 12 months?
Hearing	Hearing, cannot hear at all or use a hearing aid?	Deafness or partial hearing loss
Vision	Seeing, cannot see at all or wear glasses or contact lenses to assist their vision?	Blindness or partial sight loss
Communication	Speaking or making themselves understood, cannot speak at all or use aids or specialised equipment to assist them to communicate? Do not include children who cannot yet speak unless there is a specific problem.	Communication difficulty (a difficulty with speaking or making yourself understood)
Mobility	Mobility difficulties for example moving about, walking, climbing stairs; are not mobile at all or use specialised equipment or have personal support services such as a home help or personal assistant to help them to be mobile?	A mobility or dexterity difficulty (a condition that substantially limits one or more basic physical activities such as walking, climbing stairs, lifting or carrying)
Dexterity	Dexterity difficulties (by that I mean lifting, carrying, grasping or holding objects); cannot lift, carry, grasp or hold at all; or use specialised equipment to help them with these actions?
Learning difficulty/disability	A difficulty learning for example at school, college, work or in other places. This may be due to a condition such as dyslexia or Attention Deficit Hyperactivity Disorder or it may not have a name.	A learning difficulty, an intellectual difficulty, or a social or behavioural difficulty
	An intellectual difficulty or developmental delay. This may not have a name but include things like Down's syndrome, autism, Fragile X Syndrome and other conditions.
	A social or behavioural difficulty, for example difficulty making friends or aggressive outbursts etc. These may be associated with conditions such as autism, Attention Deficit Hyperactivity Disorder, Asperger’s Syndrome or may have no apparent cause or name.
Pain or discomfort	Long-term pain or discomfort that is always present or reoccurs from time to time or take medication to manage any long-term pain or discomfort?	Long-term pain or discomfort
Shortness of breath	Shortness of breath or difficulty breathing or use specialised equipment such as a nebuliser, oxygen concentrator or cylinder or ventilator to assist with breathing?	Shortness of breath or difficulty breathing (such as asthma)
Confusion	Frequent periods of confusion or difficulty remembering things? These difficulties may be associated with diseases such as Alzheimer's, dementia or as a result of a brain injury or stroke?	Frequent periods of confusion or memory loss
Long-term health conditions	Any of the following long-term conditions that have lasted or are expected to last 6 months or more and that have been diagnosed by a health professional: asthma or severe allergies; heart condition or disease; kidney condition or disease; cancer; diabetes; epilepsy; Cerebral Palsy; Spina Bifida; Cystic Fibrosis; muscular dystrophy; multiple sclerosis; migraines; paralysis of any kind; missing or malformed arms, legs, fingers or toes; complex medical care needs; or other.	A chronic illness (such as cancer, HIV, diabetes, heart disease or epilepsy)
Brain damage	Any difficulty carrying out everyday activities as a result of a head injury, stroke or any other sort of brain damage?	NA
Other conditions	Any other difficulties or limitations because of a physical condition, mental health condition or health problem that we have not already covered?	Other condition
No conditions	NA	No condition

### Data analysis

First, we compared the 2007 NISALD and the 2011 Census in relation to the distribution of age, sex, region using Nomenclature of Territorial Units for Statistics (NUTS), and disability (both in terms of distinct disabilities categories and the overall number of health conditions reported). To overcome the complex issue of comparing the 2007 NISALD (sample) with the 2011 Census (population), standardised difference scores were computed using the *stddiffi* command in Stata 15 [17] to test for differences in relation to key socio-demographic and disability characteristics between the two data sources. Unlike other statistical tests (e.g. chi-square), the standardised difference score is not influenced by sample size [18], and can be more informative than p-values for comparing across data sources that differ in relation to sample size [19]. Standardised differences of 0.2, 0.5, and 0.8 represent small, medium, and large standardised differences respectively [20]; standardised difference scores of less than 0.1 suggests no meaningful differences between data sources in relation to the distribution of the variable under consideration [21].

Second, we estimated and compared the prevalence and 95% confidence intervals of any learning difficulty, intellectual difficulty, and/or social-behavioural difficulty across the two data sources. To achieve comparability with the 2011 Census, a binary variable was generated for the 2007 NISALD data to represent endorsement/experiences across the three individual categories (i.e., any learning difficulty, intellectual difficulty, and/or social/behavioural difficulty). These two stages of analyses included all individuals living in private households who participated in the Census (N=1,702,217) and all adults/children who completed the 2007 NISALD screening interview (N=23,689), not just those who met the disability criteria for completing the main questionnaire.

Third, we recorded the prevalence estimates separately for any learning difficulty, any intellectual difficulty, and any social/behavioural difficulty for the NISALD only (comparable data in 2011 Census was not available).

Fourth, we compared the socio-demographic and health-related characteristics of individuals reporting any learning difficulty, intellectual difficulty and/or a social or behavioural difficulty (LD) in NISALD compared to the Census.

Finally, given that the 2007 NISALD contained an indicator that more explicitly identified those with intellectual disability (comparable data not available in the Census), we examined the socio-demographic and health characteristics associated with endorsement of this indicator.

## Results

### Comparability of 2007 NISALD and 2011 Census

The 2007 NISALD and the 2011 Census had similar distributions for sex, age, and region; standardised difference scores for these comparisons were all <0.10 (see Table 2) suggesting no meaningful differences in the distribution of these characteristics between the two data sources.

The medium effect size of >0.5 for number of health conditions indicates that the 2007 NISALD and the 2011 Census differed considerably in relation to the number of health conditions experienced. Specifically, the 2007 NISALD had a higher percentage of individuals with no disability/health conditions and a higher percentage of individuals with multiple comorbid health conditions, when compared to the 2011 Census.

**Table 2: Comparison of the sex, age, geographical, and health-related distribution of the general population sample recruited in the 2007 Northern Ireland Survey on Activity Limitations and Disability compared to the 2011 Northern Ireland Census.** 

Socio-demographic and health characteristics		2007 NISALD		2011 Census		Standardized differences
		All (N=23,689)		All (N=1,702,217)	
		N	%	N	%
Sex	Male	11,492	48.5	827,932	48.6	0.002
	Female	12,190	51.5	874,285	51.4
Age (years)	0-15 years	5,192	21.9	355,430	20.9	0.066
	16-24 years	3,176	13.4	202,794	11.9
	25-34 years	2,972	12.6	228,377	13.4
	35-44 years	3,506	14.8	239572	14.1
	45-54 years	3,102	13.1	240,001	14.1
	55-64 years	2,628	11.1	188,582	11.1
	65-74 years	1,860	7.9	141,259	8.3
	75+ years	1,222	5.2	106,202	6.2
Region	Belfast	3,422	14.4	254,631	15.0	0.034
	Outer Belfast	5,135	21.7	371,176	21.8
	East	5,666	23.9	420,515	24.7
	North	3,862	16.3	267,788	15.7
	West/South	5,604	23.7	384,315	22.6
Number of health conditions	None	19,513	82.3	1,168,020	68.7	0.510
	One	816	3.4	297,950	17.5
	Two	912	3.9	103,200	6.1
	Three	757	3.2	67,135	3.9
	Four or more	1,691	7.2	65,912	3.8

### Prevalence of learning difficulty, intellectual difficulty and/or social/behavioural difficulty in the general population

The prevalence of experiencing any learning difficulty, intellectual difficulty, and/or social/behavioural difficulty in the NISALD was lower than that obtained via the 2011 Census (1.6% vs. 2.0%). Of the three conditions, based on the NISALD data alone (comparable estimates for the 2011 Census were not available), the prevalence was highest for any learning difficulty, followed by social or behavioural difficulty, and then intellectual difficulty (see Table 3).

**Table 3: Comparison of the prevalence of learning difficulty, intellectual difficulty and/or a social or behavioural difficulty (LD) in the general population of Northern Ireland: data from the 2007 Northern Ireland Survey on Activity Limitations and Disability and the 2011 Northern Ireland Census:** 

Disability		2007 NISALD		2011 Census	
		Overall (N=23,689)		Overall (N=1,770,217)	
		0-15 years (n=5,172)		0-15 years (n=355,430)	
		16 years + (n=18,517)		16 years + (n=1,346,787)	
		n	Prevalence (95% CI)	n	Prevalence
A learning difficulty, intellectual disability and/or a social or behavioural difficulty	Overall	389	1.6% (1.5-1.8)	34,401	2.0%
	0-15 years	186	3.6% (3.1-4.1)	13,530	3.8%
	16 years +	203	1.1% (.9-1.3)	20,871	1.5% 5
Any intellectual difficulty	Overall	130	0.5% (0.4-0.7)	NA	NA
	0-15 years	67	1.3% (1.0-1.6)	NA	NA
	16 years +	63	0.3% (0.3-0.4)	NA	NA
Any learning difficulty	Overall	298	1.3% (1.1-1.4)	NA	NA
Any social or behavioural difficulty	Overall	176	0.7% (0.6-0.9)	NA	NA

### Correlates of learning difficulty, intellectual difficulty, and/or social/behavioural difficulty in the general population

As presented in Table 4, there was little difference in the sex distribution of any learning difficulty, intellectual difficulty and/or social/behavioural difficulty between the 2007 NISALD and the 2011 Census (standardised difference score <0.01), with approximately 70% of those satisfying this indicator being male.

Small-to-medium effect sizes were evident for the other characteristics. Specifically, a higher percentage of younger people (0-15 years) with any learning difficulty, intellectual difficulty and/or social/behavioural in NISALD compared to the Census. Higher percentages of individuals with any learning difficulty, intellectual difficulty and/or social/behavioural difficulty in NISALD lived outside the Belfast/Outer Belfast area, were not economically active, and had a greater number of health conditions, compared to those in the 2011 Census.

**Table 4: Comparison of the socio-demographic and health-related characteristics of individuals reporting any learning difficulty, intellectual difficulty and/or a social or behavioural difficulty (LD) in the general population of Northern Ireland: data from the 2007 Northern Ireland Survey on Activity Limitations and Disability and the 2011 Northern Ireland Census:** 

		Learning difficulty, intellectual difficulty and/or a social or behavioural difficulty (LD)					Standardized differences
		2007 NISALD		2011 Census			
		(n=389)		(n=34,401)			
		
		N	%	N	%		
Sex	Male	260	66.8	23,108	67.2	0.007
	Female	129	33.2	11,293	32.8
Age	0-15 years	186	47.8	13,530	39.3	0.247
	16-24 years	58	14.9	6,800	19.8
	25-34 years	26	6.7	3,880	11.3
	35-44 years	45	11.6	3,260	9.5
	45-54 years	37	9.5	3,230	9.4
	55+years	36	9.5	3,701	10.7
Region*	Belfast	50	12.9	6,333	18.4	0.181
	Outer Belfast	77	19.8	6,897	20.0
	East	90	23.1	7,959	23.1
	North	80	20.6	5,564	16.2
	West/South	92	23.6	7,379	21.4
Economic activity	Active	40	10.3	5,751	16.7	0.312
	Inactive	127	32.6	13,609	39.6
	Other	36	9.3	1,511	4.4
	Not working age	186	47.8	13,530	39.3
General health*	Very good	92	23.7	7,456	21.7	0.174
	Good	115	29.6	11,505	33.4
	Fair	76	19.5	9,906	28.8
	Bad	33	8.5	3,796	11.0
	Very bad	16	4.1	1,738	5.1
Number of health conditions	One	73	18.8	12,526	36.4	0.444
	Two	84	21.6	7,462	21.7
	Three	67	17.2	5,277	15.3
	Four or more	165	42.4	9,136	26.6

### Socio-demographic and health-related characteristics of intellectual difficulty

Finally, we focused on the 2007 NISALD data separately to identify the characteristics of individuals reporting any intellectual difficulty (see Table 5 for prevalence and 95% confidence intervals). The majority of individuals surveyed with an intellectual difficulty (overall and in both age categories) were male, living outside Belfast and the Outer Belfast area, had a severe intellectual difficulty, had multiple comorbid health conditions (communication difficulties were highly prevalent, as were learning difficulties and other social/behavioural difficulties) but, in general, self-reported that their health was good or very good. A higher proportion of those age 16 years or over resided in the East or West and South NUTS regions of Northern Ireland and reported difficulties with vision, chronic illness and head injury compared to those aged 0-15 years. A higher proportion of those age 0-15 years resided in the Outer Belfast or North NUTS regions of Northern Ireland and reported social or behavioural difficulties compared to those aged 16 years or over.

**Table 5: Socio-demographic and health-related characteristics of individuals reporting any intellectual difficulty in NISALD (n=130):** *Note. Percentages do not total 100 due to missing data

		Prevalence (95% CI)		
Socio-demographic characteristics		Overall	0-15 years	16 years +
Age	0-15 years	51.5% (42.6-60.1%)	-	-
	16-24 years	20.0% (13.5-27.9%)	-	-
	25-34 years	6.9% (3.2-12.7%)	-	-
	35-44 years	6.2% (2.7-11.8%)	-	-
	45-54 years	12.2% (7.2-19.2%)	-	-
	55+ years	3.1% (0.8-7.7%)	-	-
Sex	Male	69.2% (60.5-77.0%)	70.1% (57.7-80.7%)	68.3% (55.3-79.4%)
	Female	30.8 (23.0-39.5%)	29.9% (19.3-42.3%)	31.7% (20.6-44.7%)
Region	Belfast	6.9% (3.2-12.7%)	7.5% (2.5-16.6%)	6.3% (1.8-15.5%)
	Outer Belfast	20.0% (13.5-27.9%)	22.4% (13.1-34.2%)	17.5% (9.1-29.1%)
	East	26.9% (19.5-35.4%)	22.4% (13.1-34.2%)	31.7% (20.6-44.7%)
	North	16.9% (10.9-24.5%)	20.9% (11.9-32.6%)	12.7% (5.6-23.5%)
	West and South	29.3% (21.6-37.8%)	26.9% (16.8-39.1%)	31.7% (20.6-44.7%)

Disability and other health-related characteristics				

Severity of intellectual disability	Mild	6.2% (2.7-11.8%)	3.0% (0.4-10.4%)	9.5% (3.6-19.6%)
	Moderate	28.4% (20.9-37.0%)	34.3% (23.2-46.9%)	22.2% (12.7-34.5%)
	Severe	65.4% (56.5-73.5%)	62.7% (50-74.2%)	68.3% (55.3-79.4%)
General health*	Very good	27.4% (19.5-36.6%)	33.3% (21.7-46.7%)	20.8% (10.8-34.1%)
	Good	43.4% (34.1-53.0%)	40.0% (27.6-53.5%)	47.2% (33.3-61.4%)
	Fair	24.8% (17.1-33.8%)	23.3% (13.4-36.0%)	26.4% (15.3-40.3%)
	Bad/Very bad	4.4% (1.5-10%)	3.3% (0.4-11.5%)	5.7% (1.2-15.7%)
Health conditions (any)	Sight	10.0% (5.4-16.5%)	4.5% (0.9-12.5%)	15.9% (7.9-27.3%)
	Hearing	11.5% (6.6-18.3%	10.4% (4.3-20.3%)	12.7% (5.6-23.5%)
	Communication	50.0% (41.1-58.9%)	47.8% (35.4-60.3%)	52.4% (39.4-65.1%)
	Mobility	23.1% (16.1-31.3%)	20.9% (11.9-32.6%)	25.4% (15.3-37.9%)
	Dexterity	29.2% (21.6-37.8%)	25.4% (15.5-37.5%)	33.3% (22.0-46.3%)
	Pain	8.5% (4.3-14.6%)	7.5% (2.5-16.6%)	9.5% (3.6-19.6%)
	Chronic Illness	45.6% (36.6-54.3%)	41.8% (29.8-54.5%)	49.2% (36.4-62.1%)
	Breathing	10.8% (6.0-17.4%)	14.9% (7.4-25.7%)	6.3% (1.8-15.5%)
	Learning difficulty	70.7% (62.2-78.4%)	68.7% (56.2-79.4%)	73.0% (60.3-83.4%)
	Social or behavioural difficulty	53.7% (44.1-61.8%)	62.7% (50.0-74.2%)	42.9% (30.5-56.0%)
	Memory	15.4% (9.7-22.8%)	10.4% (4.3-20.3%)	20.6% (11.5-32.7%)
	Emotional, psychological or mental health condition	20.0% (13.5-27.9%)	19.4% (10.8-30.9%)	20.6% (11.5-32.7%)
	Head Injury	3.8% (1.3-8.7%)	1.5% (0.00-8.0%)	6.3% (1.8-15.5%)
Number of health conditions	One	3.8% (1.3-8.7%)	4.5% (0.9-12.5%)	3.2% (0.4-11.0%)
	Two	15.4% (9.7-22.8%)	13.4% (6.3-24.0%)	17.5% (9.1-29.1%)
	Three	20.0% (13.5-27.9%)	22.4% (13.1-34.2%)	17.5% (9.1-29.1%)
	Four or more	60.7% (51.8-69.2%)	59.7% (47.0-71.5%)	61.9% (48.8-73.9%)

## Discussion

### Survey comparisons of sex, age, geographic, and health-related distributions

Relating to sex, age and geographic location, the NISALD delivered an accurate and reliable representation of the Northern Ireland population (as enumerated in the 2011 Census). Standardised difference scores for these comparisons were all <0.10. A key advantage of national survey programmes, such as NISALD, is that samples are carefully constructed to be statistically representative of the whole population and quality control is generally of a high standard [22]. The representativeness of the NISALD data therefore, based on these key demographic characteristics, was not unexpected. However, there was a notable difference in relation to the distributions of number of health conditions between data sets. A higher proportion of the NISALD sample (82.3%) indicated an absence of household disability compared with Census household data (disability absence = 68.7%). A higher proportion of the NISALD sample also reported the presence of four or more disabilities (7.2%) compared with Census household data (>4 disabilities = 3.8%). While the higher degree of specificity of health condition classification in the NISALD may have accounted for these differences, there is evidence that the distribution of health status responses may be influenced by mode of administration [23, 24]. For instance, it may have been the case that the crude self-report in the Census was not picking up the more serious health conditions that the detailed face-to-face/telephone interview was capturing by providing respondents with an opportunity for clarification. This means that the NISALD might have obtained a more complete and accurate distribution of health limitations when compared to the Census.

### Intellectual disability prevalence estimate comparisons between the 2011 Census data and the NISALD data

Satisfied that the data from both sources could be reliably compared, our findings indicated that intellectual disability, as crudely approximated by the recoded 2007 NISALD data and the 2011 Census data, returned an overall prevalence rate of 1.6 – 2%. For those aged 0-15 years, prevalence estimates were 3.6 and 3.8% respectively, and for those aged 16 years or older, prevalence estimates were 1.1 and 1.5% respectively.

While these estimates were within range of some internationally cited population prevalence estimates of 1-3% [25, 26] they were generally above those cited in the most recent meta-analysis of international intellectual disability prevalence estimation by McKenzie et al. (0.05 – 1.55%) [1]. However, extricated from ‘learning difficulty’ and ‘social or behavioural difficulty’, intellectual disability alone returned an overall prevalence rating of 0.5% (0-15 years = 1.3%; 16 years or over = 0.3%) which was more consistent with this meta-analysis estimate range. More importantly however, these estimates were consistent with those derived from studies in the meta-analysis that employed survey questions as the mode of intellectual disability classification (estimates ranged from -0.2 - 1%). While a high proportion of these individuals also recorded the presence of a learning difficulty (70%) or a social or behavioural difficulty (54%), these data suggested that, when defined and distinguished from other forms of disability, intellectual disability specifically in Northern Ireland, as recorded within private households, may be less prevalent than has previously been suggested/reported [27].

Notably, this 0.5% prevalence estimate was also consistent with estimates derived from other Northern Ireland administrative data sources (e.g. Northern Ireland GP register data, Northern Ireland Health & Social Care Trust data, Northern Ireland Housing Executive data) [28] and with the most widely used estimate of the number of adults in England known to have an intellectual disability [29]. Derived from GP registers in the National Health Service (NHS), 0.5% (n=206,132) of patients were identified as having an intellectual disability in England in 2013 (N=53.3m) (these data however were restricted to individuals over 18 years of age, were under-representative of people with mild learning disabilities and included those living in residential settings – three important distinctions from the NISALD data).

Using the 2011 Census general population base (i.e. N=1,770,217) and the prevalence of intellectual disability of 0.5% (95%CI 0.4-0.7%), a crude approximation (based on 2011 Census general household population figures and an unchanged prevalence of intellectual disability between 2007 and 2011) is that between 7,081 and 12,392 individuals living in private households in Northern Ireland met the NISALD criterion for an intellectual disability in 2011. Given the limitations of the NISALD data (detailed in limitations section below) and the many barriers and obstacles individuals with intellectual disability commonly face in accessing timely, appropriate and effective health care [30-38] we believe the estimates derived from our data analysis and the English GP register data in 2013, while consistent, are likely to be underestimates.

### Socio-demographic and health-related characteristics of those individuals who endorsed the more specific intellectual difficulty item from the 2007 NISALD survey

Using the cruder classification of intellectual disability from the Census, it was clear that the ID subsample distributions of age and geographical location, shown to be comparable at an overall population level, were, in this context, statistically different. In fact, sex was the only variable that did not statistically differ between intellectual disability subsamples when the data was framed using the classification of intellectual disability from the Census. While the differences in general health between subsamples were negligible, notable disparities between the number of health conditions were evident. Lin and colleagues [5], demonstrated how more precise classification of intellectual disability resulted in the identification of higher rates of comorbidity. While the cruder classification of intellectual disability in this analytic step may have affected comorbidity comparisons between data sources, it would seem logical that the greater level of specificity in each of the other NISALD health conditions also influenced findings.

### Intellectual disability subsample comparisons of sex, age, geographical, economic-activity and health-related distributions from both surveys, as derived from the 2011 Census and NISALD Census-comparator items.

Using the explicit intellectual disability indicator from the NISALD data it was possible to more accurately locate the regions in Northern Ireland where individuals with intellectual disability live and to describe Northern Ireland’s intellectual disability population in relation to several important socio-demographic and health related characteristics. Compared to the NISALD measure of intellectual disability, the 2011 Census recorded a larger proportion of the population with intellectual disability in two of the five NUTS regions and a lower proportion of intellectual disability in the remaining three regions. Notable differences were evident in relation to Belfast (NISALD intellectual disability =6.9% versus Census ‘11=15%) and in relation to Western and Southern Northern Ireland (NISALD ID=29.3% versus Census ’11=22.6%). The NISALD intellectual disability indicator also returned a much younger age profile and suggested a more severe morbidity/comorbidity profile compared to the 2011 Census data.

While a minimum prevalence rate of 10% was evident for all but one (long-term pain) of the 13 health conditions, communication difficulties (50%), and chronic illness (45.6%) (in addition to learning difficulty and social and behavioural difficulty) were particularly prevalent. These rates are not unexpected. Poor communication is recognized as a significant barrier for people with intellectual disability [39-42] while increased longevity amongst the intellectual disability population has led to a rise in secondary conditions such as obesity and Type 2 diabetes as well as an increased prevalence in a number of chronic illnesses [43-45].

A notable advantage of the NISALD was its supplementary data on disability severity. While distinguishing intellectual disability from learning difficulties and social and behavioural difficulties is critical for defining, locating and responding to intellectual disability effectively, qualifiers of ‘mild’, ‘moderate’ and ‘severe’ are commonly employed by health care practitioners to indicate the level of support that is required by individuals with varying levels of intellectual impairment [46]. NISALD therefore afforded an opportunity to recognise that over 65% of those living with an intellectual disability in Northern Ireland experienced severe impairment.

These findings clearly highlight the importance of definition and specificity in the detection and classification of intellectual disability in the general population. They also reveal the value of supplementing Census data with high quality national survey data. Given that household probability survey sampling will almost certainly under detect phenomena such as intellectual disability, it can be expected that the prevalence estimate of 0.5% reported here will fall short of the Census household prevalence rate in 2021 (although census returns may also under-report).

### Limitations

Several important limitations must be acknowledged in relation to the proposed findings. First and most importantly, the current set of analyses were restricted to people living in private households. This means that individuals in residential care were excluded. Given their residential status we also suspect that our findings will be heavily weighted towards younger people with intellectual disability.

Second, as is commonly the case with national survey programmes and the investigation of low prevalence phenomena, the number of people with intellectual disability in the NISALD data (n=130) limited our analyses e.g. half of the intellectual disability sample was under 15 years of age therefore more in-depth analyses of e.g. health indicators and economic activity known to be age variant, were restricted.

Third, while the NISALD data offered a more explicit measure of intellectual disability, compared to the 2011 Census, it was not without its own definition complications. The NISALD intellectual disability item explicitly referred to an intellectual ‘difficulty’ rather than ‘disability’ and included autism as an exemplar condition which was also included as a condition under the social or behavioural difficulty item. The precise interpretation of this intellectual disability indicator by respondents therefore was unknown and may have influenced item endorsement.

Fourth and finally, as identified by Murphy [28], the 2007 NISALD and the 2011 Census are only two data sources that provide information on the landscape of intellectual disability in Northern Ireland. Ultimately, it is important to strive towards collating and combining all available and relevant information at a regional-level to ensure that prevalence estimates of intellectual disability in Northern Ireland are as accurate as possible. Indeed, there has been a surge in the use of the ‘capture-recapture method’ in epidemiological research in recent years for this specific purpose [47]. Although not without limitations [48], capture-recapture techniques have been used in attempts to estimate or adjust for the extent of incomplete ascertainment of cases (e.g. for specific health conditions or diseases such as alcohol use problems [49], using information from overlapping lists of cases derived from distinct sources. Whilst this method may well offer a potentially useful avenue for future intellectual disability research in Northern Ireland, the potential utility of the capture-recapture method would largely depend on gaining access to data relating to the prevalence of intellectual disability from multiple different government and community/voluntary services and agencies (e.g. GP registers, Health and Social Care Trust data; community day service attendance; residential care homes and sheltered accommodation), which has historically proven difficult in an Northern Ireland context.

### Preparing for the future

The future import and potential of these sources of data for informing the assessment of need of people with intellectual disabilities, healthcare planning and government policy in Northern Ireland cannot be understated. The United Nations Convention on the Rights of People with Disabilities requires States Parties to commit to the collection of appropriate information, including statistical and research data to enable them to formulate and implement policies to give effect to the Convention [50]. According to Glover [22] ‘if an issue is both visible and quantifiable in official statistics, it becomes much harder for public bodies locally and nationally to ignore.’ (p.15). In other countries, data linkage approaches have provided the basis for the type of service use and needs assessment work that is fundamental to reviewing healthcare policy [e.g. 51, 52]. Once Northern Ireland’s new and improved Census 2021 intellectual disability indicator comes ‘on line’ [53], citizens (with the support of carers/family members where necessary) will be afforded the opportunity to officially and specifically record the presence of ‘*An intellectual or learning disability (for example Down syndrome)*’. Moreover, specific separate options to also record ‘*A learning difficulty (for example dyslexia)*’ and/or ‘*Autism or Asperger syndrome*’ will further enhance the specificity and improve the measurement of intellectual disability in the next Northern Ireland Census. A variety of other administrative data resources will also have the potential to unlock the full policy potential of this more explicit and specific intellectual disability measure (e.g. national mortality statistics, health service data, education data).

## Conclusion

A burgeoning international research literature continues to detail the extreme disadvantages that are disproportionately faced by those in society living with an intellectual disability. Worryingly, this extreme population-specific disadvantage is further and significantly compounded by the fact that those living with intellectual disability, in many countries, remain unseen. Intellectual disability specifically, at a population level, has either remained unrecorded and undetected or has been camouflaged, hidden, or buried within general health data, that have referred to limitations in day-to-day activities or inability to work as a result of health problems or disability. We hope that these findings will highlight the availability, utility and import of intellectual disability data in Northern Ireland, and promote and stimulate future use of this data in the region. We also hope that these findings will incentivise those in power to facilitate Northern Ireland data custodians to share/link available intellectual disability relevant data where possible. Finally, we believe that the current findings lay some useful foundations for the more advanced and sophisticated intellectual disability statistical modelling that will be possible in the years to come and the policy changes that will emerge as a consequence.

## Ethics Statement

Ethical approval for this study was issued by Ulster University’s Research Governance - Reference Number 16/0032 and the Administrative Data Research Network (Re: PROJ-099 – Learning Disability and Northern Ireland: Achieving Proportionate Universalism through Administrative Data Research).
